# Early post-operative NI-RADS predicts recurrence and survival in high-risk oral cavity squamous cell carcinoma undergoing adjuvant radiotherapy

**DOI:** 10.1007/s11547-025-02121-9

**Published:** 2025-10-27

**Authors:** Mariangela Massaccesi, Marco Panfili, Rosalinda Calandrelli, Silvia Longo, Francesco Pastore, Francesco Miccichè, Calogero Casà, Stefano Settimi, Dario Antonio Mele, Nicola Dinapoli, Ciro Mazzarella, Simona Gaudino, Luca Tagliaferri, Jacopo Galli, Maria Antonietta Gambacorta, Giovanni Almadori

**Affiliations:** 1 Department of Radiation Oncology, Fondazione Policlinico Universitario Agostino Gemelli IRCCS, Rome, Italy; 2 Department of Neuroradiology, Fondazione Policlinico Universitario Agostino Gemelli IRCCS, Rome, Italy; 3 U.O.C. Di Radioterapia Oncologica, Fatebenefratelli Isola Tiberina, Gemelli Isola, 00186 Rome, Italy; 4 Unit of Otorhinolaryngology, “A. Gemelli” University Hospital Foundation IRCCS, Rome, Italy; 5 Catholic University of the Sacred Heart, Rome, Italy; 6 Fondazione Policlinico Universitario Agostino Gemelli IRCCS, Largo Agostino Gemelli, 8, 00168 Rome, Italy; 7 Head, Neck and Sensory Organs Department, Catholic University of Sacred Heart, Rome, Italy

**Keywords:** Oral cavity squamous cell carcinoma, Neck imaging reporting and data systems, Post-operative radiotherapy, Recurrence, Survival

## Abstract

**Purpose:**

To evaluate the prognostic value of the Neck Imaging Reporting and Data Systems (NI-RADS) in early post-operative imaging for predicting recurrence and survival outcomes in high-risk oral cavity squamous cell carcinoma (SCC) patients undergoing post-operative radiotherapy (PORT).

**Methods:**

This retrospective study included 84 patients with high-risk oral cavity SCC who were scheduled for PORT after radical surgery between January 2013 and May 2024. Early imaging with contrast-enhanced CT or MRI was performed within 12 weeks post-surgery and scored using the NI-RADS system. Associations between NI-RADS scores, recurrence, and survival outcomes were analyzed using Kaplan–Meier and Cox proportional hazards models.

**Results:**

Although NI-RADS was originally designed for post-treatment surveillance, we applied it to early post-operative imaging as an exploratory risk-stratification tool. NI-RADS scores significantly predicted regional disease-free survival (DFS) and overall survival (OS). Patients with higher NI-RADS *T* and *N* scores had poorer outcomes. Multivariable analysis confirmed early NI-RADS *T* as an independent predictor of OS (*p* = 0.01). Interobserver agreement for NI-RADS classifications was strong (Weighted Kappa: *T* = 0.837, *N* = 0.855). Although higher radiotherapy doses were administered to patients with NI-RADS 2–3 scores, these patients demonstrated worse outcomes, reflecting aggressive disease.

**Conclusion:**

Early application of NI-RADS in post-operative imaging provides valuable prognostic insights, enabling risk stratification and tailored management in high-risk oral cavity SCC patients. Streamlining imaging workflows and exploring alternative therapeutic strategies for high-risk groups may further optimize outcomes.

## Introduction

Surgery remains the cornerstone of oral cavity cancer treatment. However, in patients with high-risk pathological features—such as advanced *T* stage (*T*3/*T*4), positive or close margins, extranodal extension (ENE), multiple positive lymph nodes, perineural invasion, or lymphovascular space invasion—post-operative radiotherapy (PORT), often combined with chemotherapy, improves disease-free survival [1].

Despite guidelines recommending PORT within 6 weeks after surgery to limit total treatment duration to no more than 13 weeks [2–4], locoregional recurrence in oral squamous cell carcinoma (OSCC) often occurs early. As many as 28% of high-risk patients may show progression on diagnostic imaging before initiating PORT [5], underscoring the value of short-interval post-surgical imaging as advocated by the National Comprehensive Cancer Network (NCCN) [2].

Nonetheless, universal adoption of pre-PORT imaging is constrained by costs, logistics, and concerns about delaying treatment. Differentiating post-operative changes—such as surgical edema, inflammation, and reconstruction-related artifacts—from residual or recurrent disease also remains challenging [6]. Diffusion-weighted MRI may help: A meta-analysis reported that apparent diffusion coefficient (ADC) values aid differentiation between post-treatment change and recurrence in head and neck cancer [7].

The Neck Imaging Reporting and Data Systems (NI-RADS), introduced by the American College of Radiology (ACR), standardizes post-treatment imaging assessment and has shown strong predictive performance and inter-reader agreement for detecting recurrences [8–16]. However, most existing studies evaluate imaging months after curative treatment and in heterogeneous head and neck cohorts (e.g., surveillance-phase evaluations and broader HNSCC series). Although NI-RADS was designed for post-treatment surveillance rather than immediate post-operative assessment, applying it in this early window may affect classification accuracy; our use is therefore exploratory.

To address this gap, we investigated the utility of early NI-RADS scoring (within 12 weeks post-surgery) for predicting recurrence and survival in high-risk oral cavity SCC patients undergoing PORT. By clarifying how NI-RADS performs in this critical early interval, we aim to refine adjuvant management strategies and improve clinical outcomes.

## Material and methods

### Study design and participants

This retrospective observational study was conducted at Fondazione Policlinico Universitario Agostino Gemelli IRCCS, Rome, Italy, following approval from the Institutional Ethical Committee (Reference No.: 6698). It adheres to the principles of the Declaration of Helsinki and Good Clinical Practice.

Patients diagnosed with high-risk locoregionally advanced or recurrent oral cavity squamous cell carcinoma (SCC), who underwent radical surgery followed by post-operative radiotherapy (PORT) between January 1, 2013, and May 30, 2024, were included.

### Inclusion and exclusion criteria

#### Inclusion criteria

- Patients diagnosed with high-risk locoregionally advanced or recurrent oral cavity SCC scheduled for PORT after radical surgery.
- Age ≥ 18 years.
- Availability of complete medical records and imaging data.

#### Exclusion criteria

- Incomplete imaging data.
- Pre-operative chemotherapy.
- Presence of factors limiting imaging interpretation (e.g., artifacts due to movement or non-removable dental prostheses).

### Data collection

Relevant data were extracted from electronic medical records and radiology information systems. These included patient demographics (age, sex), clinical presentation, histopathological findings, imaging data (MRI and contrast-enhanced CT [CE-CT]), and follow-up outcomes. To ensure confidentiality, all data were anonymized.

#### Imaging protocol

Post-operative imaging involved MRI or CE-CT.

### MRI protocol

Performed on a 1.5 T scanner (General Electric Healthcare, Milwaukee, WI, USA) with a quadrature head–neck coil. The standard protocol included:

- Pre-contrast multiplanar T1-weighted, T2-weighted, Short Tau Inversion Recovery (STIR), and Diffusion-Weighted Imaging (DWI) sequences.
- Post-contrast 3D T1-weighted sequences after intravenous administration of paramagnetic contrast medium.

### CE-CT protocol

Conducted using a GE LightSpeed Pro 64 system (GE Medical Systems, Milwaukee, WI). Scans were performed 80 s post-intravenous administration of iodinated contrast medium, with 1.25-mm axial slice thickness and soft tissue and bone reconstruction algorithms.

### Definition of early scan

An early scan was defined as an MRI or CE-CT conducted within 12 weeks of surgery.

#### Image analysis

Two experienced neuroradiologists (MP and RC), blinded to clinical outcomes, independently reviewed the imaging studies. Discrepancies were resolved by consensus.

**Fig. 1:**
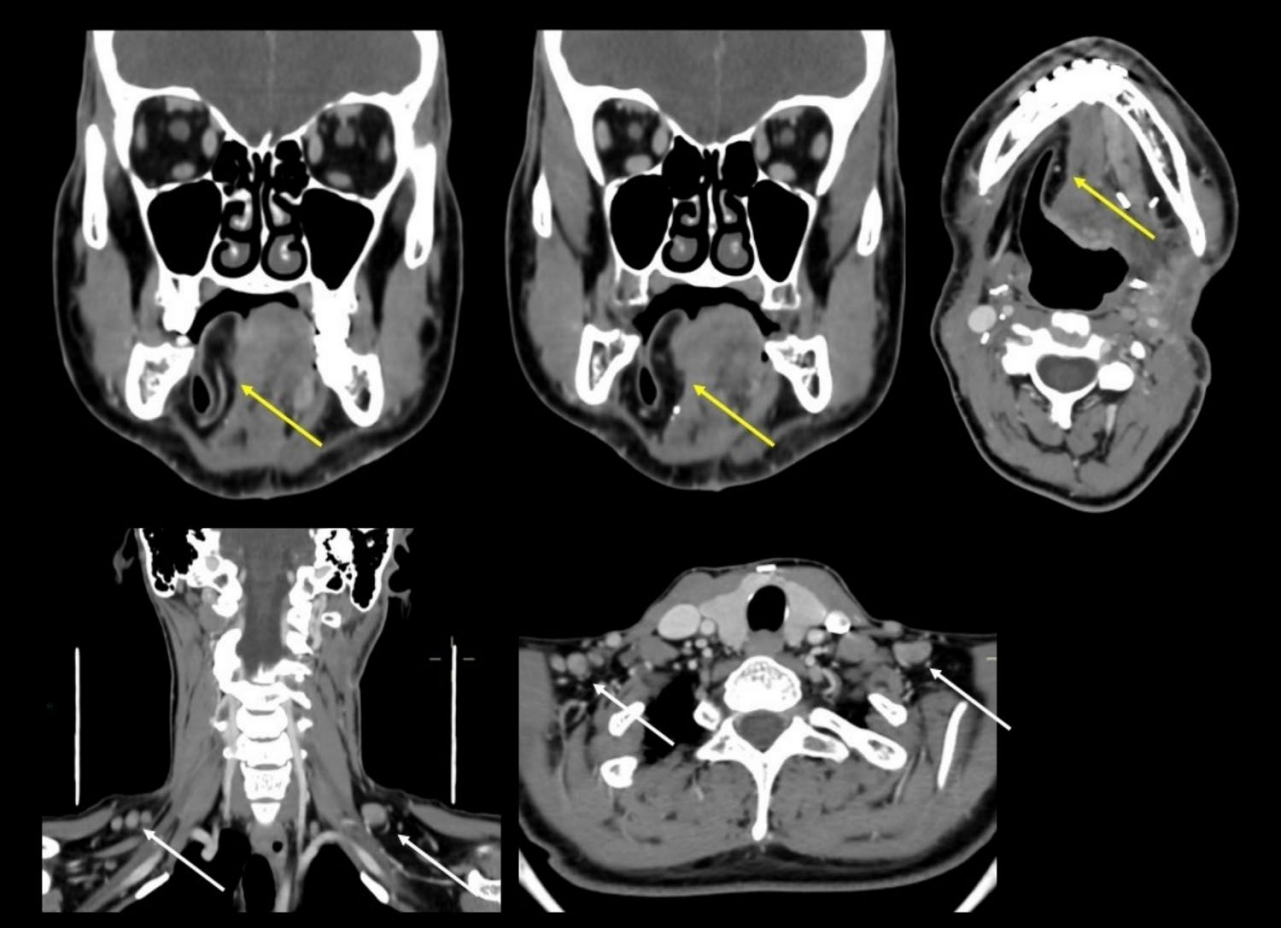
Coronal and axial post-operative contrast-enhanced CT (CE–CT) obtained 4 weeks after surgery in a patient with pT3N2c floor-of-mouth squamous cell carcinoma treated with hemiglossectomy, neck dissection, and radial forearm flap reconstruction. NI-RADS T1: no abnormal enhancement along the superficial or deep margins of the flap (yellow arrows, upper-row images). NI-RADS N2: bilaterally enlarged level Vb lymph nodes without necrosis or signs of extranodal extension (white arrows, lower-row images)

**Fig. 2:**
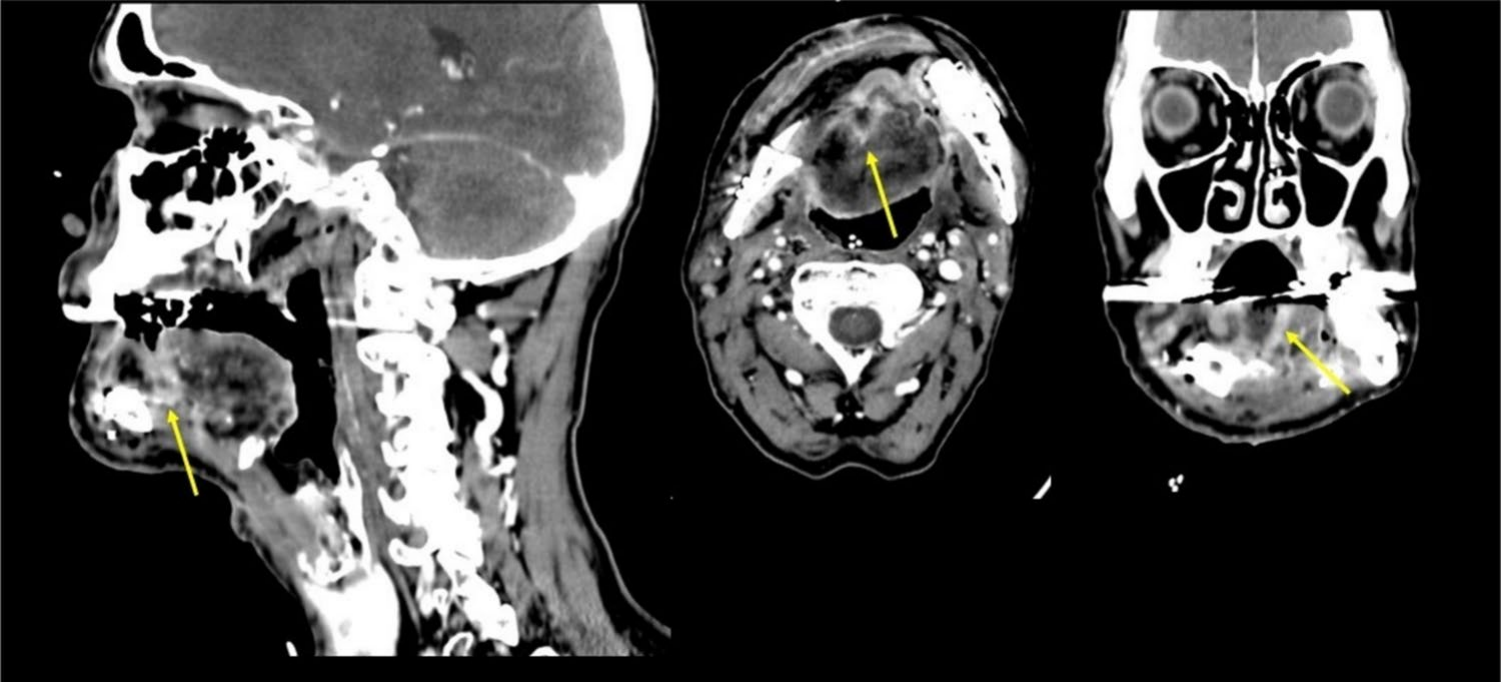
Sagittal, axial and coronal views of post-operative CE-CT after 5 weeks in a patient with pT4aN3b right inferior paramedian gingival SCC surgically treated with right emimandibulectomy, neck dissection and fibular osteo-cutaneous flap reconstruction. NI-RADS 2a for *T* supported by the presence of focal linear superficial post-contrast enhancement at the junction between the posterior margin of the adipo-cutaneous portion of the flap and the ventral tongue (yellow arrows)

**Fig. 3:**
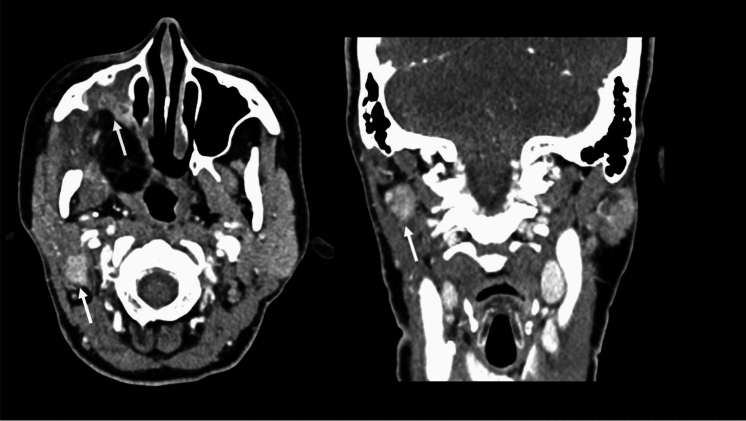
Axial and coronal views of post-operative CE-CT after 8 weeks in Patient with pT4aN3b superior right retromolar trigone SCC surgically treated with maxillectomy, neck dissection and antero-lateral thigh flap. NI-RADS 2b for *T* because there is focal ill-defined enhancement along the supero-anterior margin of the flap (yellow arrow). NI-RADS 3 for N because there is a new enlarged right retroparotid lymph node with intense enhancement and irregular margins suspicious for initial extracapsular spread (with arrow)

**Fig. 4:**
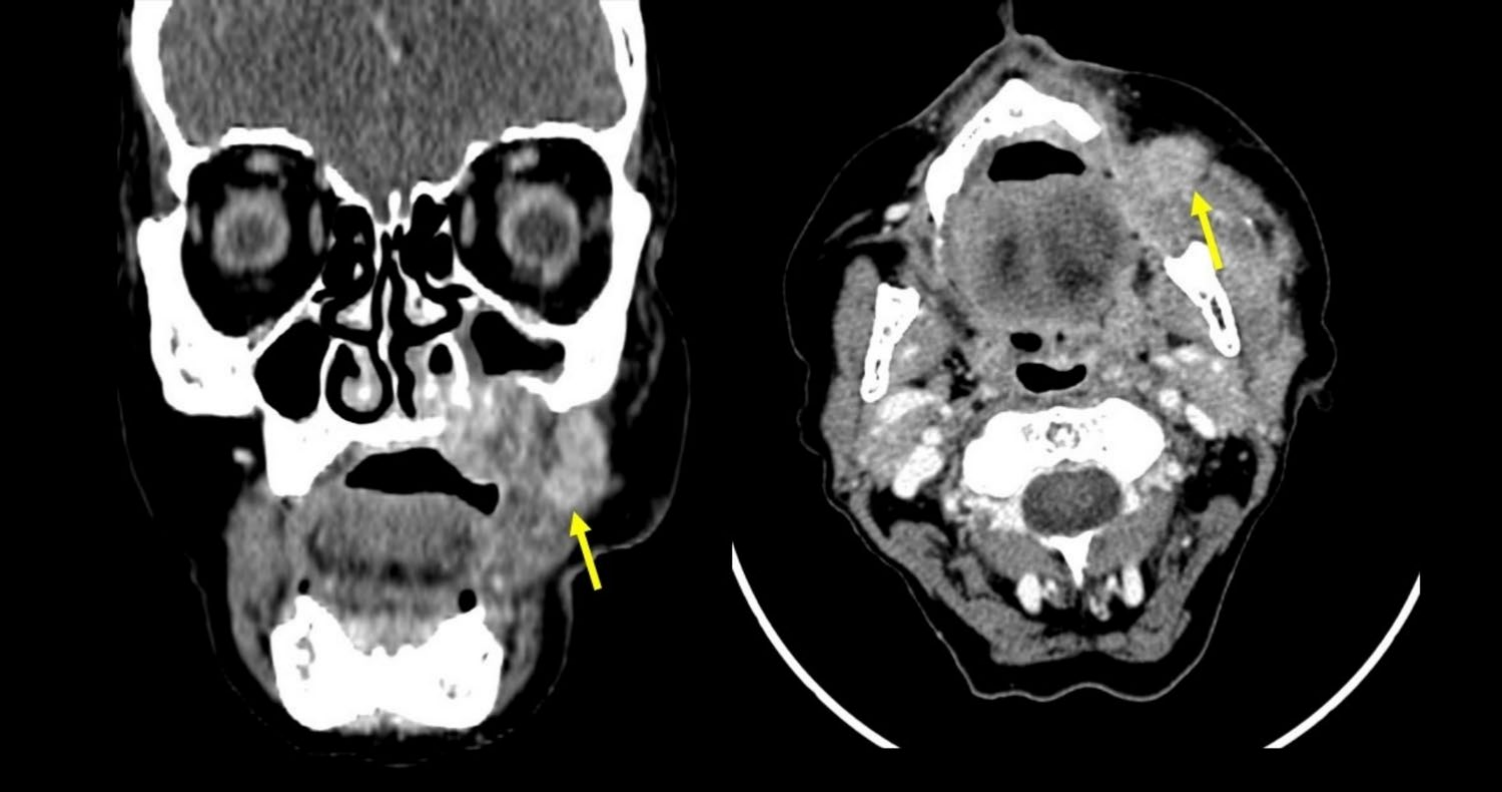
Coronal and axial views of post-operative CE-CT after 6 weeks in Patient with pT4aN1 superior left retromolar trigone SCC surgically treated with partial maxillectomy, neck dissection and temporal flap reconstruction. NI-RADS 3 for *T* because there is enhancing nodular area along the anterior margin of the flap (yellow arrow)

The Neck Imaging Reporting and Data Systems (NI-RADS) score was applied to *T* (tumor) and *N* (nodal) sites based on ACR Category Descriptors [8]. NI-RADS 1 indicated expected post-treatment changes, such as linear mucosal enhancement or non-mass-like submucosal distortion with low CT density or high T2w (not T2-intermediate) intensity signal suggesting edema/inflammation, or low T1 and T2 signal suggesting scar/fibrosis. (Fig. 1) NI-RADS 2 encompassed findings suggestive of potential recurrence, with 2a indicating focal non-mass-like mucosal enhancement or focal mucosal reduced diffusion on MRI (Fig. 2), and 2b representing deep, ill-defined non-nodular soft tissue with moderate contrast enhancement or diffusion restriction or intermediate signal on T2w MR images (Fig. 3) . For nodal sites, NI-RADS 2 described moderately enhancing residual nodal tissue or new or enlarging nodes lacking definitive abnormal features (Fig. 1). NI-RADS 3 was assigned for findings highly suspicious for recurrence, including focal discrete nodule or mass with intense contrast enhancement or MR signal characteristics similar to original tumor (Fig. 4). For nodal sites, NI-RADS 3 described residual or new or enlarging lymph nodes with necrosis, irregular borders, or extracapsular spread (Fig. 3). NI-RADS 4 denoted pathologically confirmed or unequivocal recurrent disease based on clinical or radiological evidence.

### Adjuvant radiotherapy and follow-up protocol

All cases were reviewed in a multidisciplinary tumor board to ensure tailored decision-making for each patient. Post-operative intensity-modulated radiotherapy (IMRT) was prescribed based on tumor characteristics and patient conditions, with radiation doses adjusted according to the extent of surgical margins and nodal involvement. Standard doses ranged from 60 to 66 Gy, delivered in daily fractions over six to seven weeks, while patients with unresectable residual disease received a dose boost up to 70 Gy, as determined by the tumor board. Concurrent chemotherapy was added for those with high-risk features, such as extracapsular nodal extension or positive surgical margins.

Patients were closely monitored during follow-up through regular joint visits with a radiation oncologist and ENT specialist at intervals of 3, 6, and 12 months post-treatment, followed by annual assessments. These visits included a thorough clinical evaluation with physical examination and symptom review, routine laboratory tests, and imaging studies, such as MRI or contrast-enhanced CT, to identify potential recurrences or metastases. This integrated approach facilitated comprehensive post-treatment care and early detection of disease progression.

#### Clinical endpoints

The study's primary clinical endpoint was disease-free survival (DFS), defined as the time from surgery to the first occurrence of disease recurrence or death from any cause. Secondary endpoints included overall survival (OS), locoregional control (LRC), and distant metastasis-free survival (DMFS).

### Statistical analysis

Statistical analysis included descriptive statistics to characterize the study population, with categorical variables summarized as frequencies and percentages and continuous variables reported as mean ± standard deviation. Interobserver agreement for imaging findings was assessed using the Weighted Kappa coefficient to evaluate reliability. Univariable analysis employed Kaplan–Meier survival curves and log-rank tests to evaluate the association of individual variables, such as NI-RADS scores, with recurrence and survival outcomes. Variables with *p* < 0.1 in univariable analysis were retained for multivariable analysis. Multivariable analysis used Cox proportional hazards models to identify independent predictors of recurrence and survival while adjusting for potential confounding factors. Statistical significance in all analyses was defined as *p* < 0.05. All data analyses were conducted using MedCalc Statistical Software, version 15.8 (MedCalc Software bvba, Ostend, Belgium; https://www.medcalc.org; 2015).

## Results

### Patient demographics and clinical characteristics

Between January 2013 and May 2024, a total of 149 patients with oral cavity cancer were referred to our Radiotherapy Unit for adjuvant radiotherapy. Of these, 84 patients with resected high-risk oral cavity squamous cell carcinoma (SCC) met the inclusion criteria and were enrolled in the study. The remaining 65 patients were excluded for reasons including non-SCC histologies (17 patients), lack of surgical treatment (23 patients), pre-operative chemotherapy (10 patients), prior radiotherapy (2 patients), early-stage cancer without high-risk features (3 patients), imaging artifacts from metal implants (1 patient), or unavailability of imaging data (10 patients).

The demographic and clinical characteristics of the 84 included patients are summarized in Table 1. Among them, 75 patients proceeded to receive adjuvant radiotherapy after surgery. The remaining nine patients did not undergo radiotherapy due to the presence of metastases (2 patients), poor performance status (3 patients), or personal refusal (4 patients).

**Table 1:** Patient demographics and clinical characteristics (*N* = 84)

Parameter	Category	*n*	%
Gender	Male	50	58.8
	Female	34	41.2
Age (years)	Median (range)	62 (28–89)	–
Tumor location	Tongue	41	48.2
	Buccal mucosa	18	21.2
	Floor of mouth	10	11.8
	Gingiva	15	18.8
Tumor stage	II	5	6.0
	III	18	21.4
	IVA	36	42.9
	IVB	25	29.8
Recurrences	Local	9	11.8
	Nodal	14	16.5
	Local and nodal	1	1.2
	Total recurrences	24	28.6
Histopathological grade	Well differentiated	10	12.9
	Moderately differentiated	49	57.6
	Poorly differentiated	19	22.4
	Unknown	6	7.1
Perineural invasion	Present	37	44.1
	Absent or unknown	47	55.9
Vascular invasion	Present	14	16.7
	Absent or unknown	70	83.3
Resection margin status	R0	43	51.8
	R1	27	31.8
	Close	9	10.6
	Unknown	5	5.9

Post-operative radiotherapy was delivered with a median dose of 66 Gy (range: 60–70.2 Gy) using standard fractionation (1.8–2.0 Gy per fraction). A dose boost up to 70 Gy was administered to 27 patients with suspected residual disease. Concurrent cisplatin-based chemotherapy was given to 41 patients, with a median cumulative dose of 200 mg/m^2^.

### NI-RADS categorization: distribution and interobserver agreement

Early post-operative imaging, consisting of contrast-enhanced CT (*n* = 76) or MRI (*n* = 7), was performed at a median of 6 weeks after surgery (range: 0.6–12.1 weeks). Table 2 presents the consensus results for NI-RADS classifications of *T* (tumor) and *N* (nodal) sites, as well as combined *T–N* categories.

**Table 2:** Early NI-RADS by consensus with counts and percentages (*N* = 84)

Group	Category	*n*	%
*T* category	NI-RADS T1	48	57.1
	NI-RADS T2a	16	19.0
	NI-RADS T2b	10	11.9
	NI-RADS T3	8	9.5
	NI-RADS *T* n.a.	2	2.4
N category	NI-RADS N1	66	78.6
	NI-RADS N2	7	8.3
	NI-RADS N3	11	13.1
Combined *T*–N groups	NI-RADS T1 and NI-RADS N1 (low risk)	43	51.2
	(NI-RADS T1 with NI-RADS N2) or (NI-RADS N1 with NI-RADS T2a–T2b) (intermediate risk)	24	28.6
	NI-RADS T3 or NI-RADS N3 (high risk)	17	20.2

NI-RADS = Neck Imaging Reporting and Data System; n.a. = not assessable

Fisher’s exact test revealed no significant associations between early NI-RADS *T* classification and resection margin status or pathological tumor stage. However, there was a trend toward higher radiotherapy doses for patients with higher NI-RADS *T* scores (*p* = 0.095). Similarly, early NI-RADS *N* classification was not significantly associated with extracapsular nodal extension, though higher NI-RADS *N* scores were significantly correlated with increased radiotherapy doses (*p* = 0.0013). Combined NI-RADS scores were not significantly associated with resection margin status or radiotherapy dose, though trends suggested higher scores were linked to closer or positive margins (*p* = 0.0722) and higher radiotherapy doses (*p* = 0.0952).

Because only seven patients underwent MRI, modality-specific performance could not be robustly assessed; exploratory models adjusting for imaging modality did not materially alter the direction of the NI-RADS associations but were underpowered.

Interobserver agreement for early NI-RADS classifications was high, with Weighted Kappa coefficients of 0.837 (SE 0.055, 95% CI 0.729–0.946) for *T* categories and 0.855 (SE 0.061, 95% CI 0.735–0.975) for *N* categories, indicating strong reliability between observers.

### Clinical outcomes

The median follow-up time for surviving patients was 57 months. At three years, the progression-free survival (PFS) rates for local, regional, distant, and any-site progression were 71.6%, 71.6%, 77.0%, and 53.2%, respectively. The median disease-free survival (DFS) was 51.0 months (95% CI: 15.0–62.0), while the median overall survival (OS) was 68.0 months (95% CI: 52.0–68.0).

Table 3 summarizes the univariable and multivariable analyses of factors influencing local control, regional control, distant control, any-site PFS, and OS. Conventional prognostic factors showed significant associations with outcomes. For instance, nodal tumor stage correlated with regional PFS (*p* = 0.006) and distant PFS (*p* = 0.01) in univariable analysis. Pathological tumor stage (Stage I–III vs. Stage IV) was significantly associated with distant PFS (*p* = 0.002 in univariable analysis, *p* = 0.04 in multivariable analysis) and any-site PFS (*p* = 0.0001 in univariable analysis).

**Table 3:** Univariable and multivariable analysis of factors affecting local, regional, distant, and any-site PFS, and overall survival (*N* = 84)

Factor		Local PFS		Regional PFS		Distant PFS		Any-site PFS		Overall survival	
Comparison		Univariable *p*	Multivariable *p*	Univariable *p*	Multivariable *p*	Univariable *p*	Multivariable *p*	Univariable *p*	Multivariable *p*	Univariable *p*	Multivariable *p*
Pathological primary tumor stage	pT1–pT3 vs pT4a–pT4b	n.s.	–	n.s.	–	n.s.	–	n.s.	–	n.s.	–
Pathological nodal tumor stage	pN3b vs others	n.s.	–	0.006	0.03	0.01	n.s.	0.0007	n.s.	n.s.	–
Pathological tumor stage	Stage I–III vs IV	n.s.	–	n.s.	–	0.002	n.s.	0.0001	0.04	0.1	n.s.
Presentation	Recurrence vs first diagnosis	n.s.	–	n.s	–	0.1	n.s.	n.s.	–	n.s.	–
Resection margin	Positive or close vs free	n.s.	–	n.s	–	0.03	n.s.	n.s	–	n.s.	–
Time from surgery to PORT ± chemo	≤ 9 vs > 9 weeks	n.s.	–	n.s	–	n.s	–	n.s.	–	n.s.	–
Concurrent chemotherapy	Yes vs no	n.s.	–	n.s.	–	n.s.	–	n.s.	–	n.s.	–
Radiation dose	< 70 vs 70 Gy	0.05	0.03	0.02	n.s.	0.02	n.s.	0.001	n.s.	0.0004	0.03
Early NI-RADS *T*	1 vs 2a/2b/3	0.028	0.05	0.001	n.s.	0.09	n.s.	0.02	n.s.	0.006	0.01
Early NI-RADS	1 vs 2/3	n.s.	n.s.	< 0.0001	n.s.	0.1	n.s.	0.006	n.s.	0.0008	n.s.
Early combined NI-RADS	1 vs 2/3	0.1	n.s.	0.001	n.s.	n.s	–	0.03	n.s	0.02	n.s.

PFS = progression-free survival; PORT = post-operative radiotherapy; NI-RADS = Neck Imaging Reporting and Data System; n.s. = not significant (*p* ≥ 0.05); – = not included in the multivariable model or not reported

Radiation dose also emerged as a significant factor. Patients receiving a radiation dose of 70 Gy had worse local control compared to those receiving less than 70 Gy (*p* = 0.05 in univariable analysis, *p* = 0.03 in multivariable analysis).

The NI-RADS system demonstrated strong associations with several outcomes. Early NI-RADS *T* was significantly linked to regional PFS (*p* = 0.001 in univariable analysis) and OS (*p* = 0.006 in univariable analysis, *p* = 0.01 in multivariable analysis). Similarly, early NI-RADS *N* was significantly associated with regional PFS (*p* ≤ 0.0001) and OS (*p* = 0.0008).

Because NI-RADS is an interpretive system, early post-operative inflammation and flap-related changes can yield false positives, whereas subtle early recurrences may be missed (false negatives). To contextualize clinical use in this window, we report exploratory misclassification proxies in Supplementary Table S1.

**Fig. 5:**
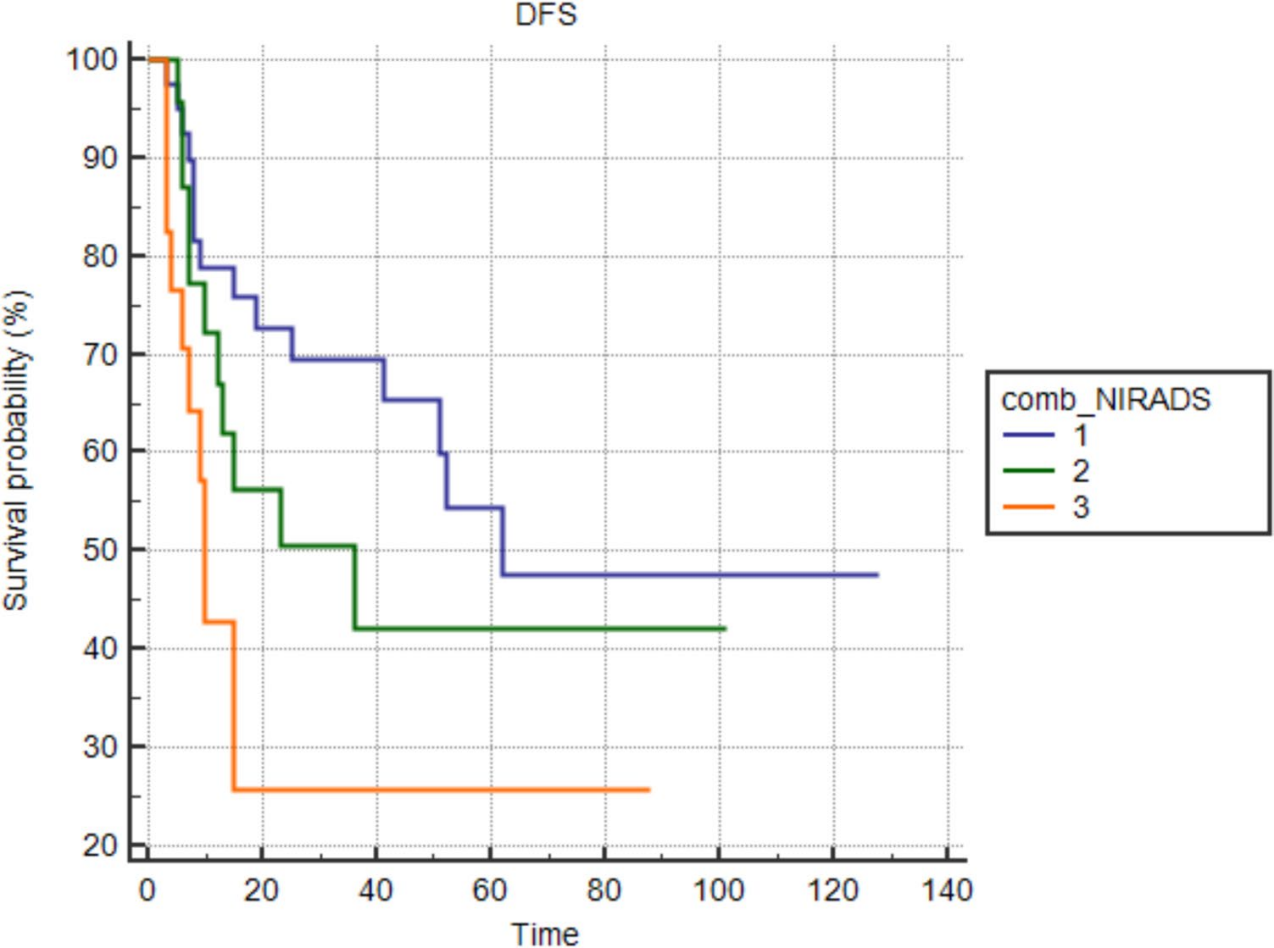
Disease-free survival (DFS) by combined NI-RADS categories

**Fig. 6:**
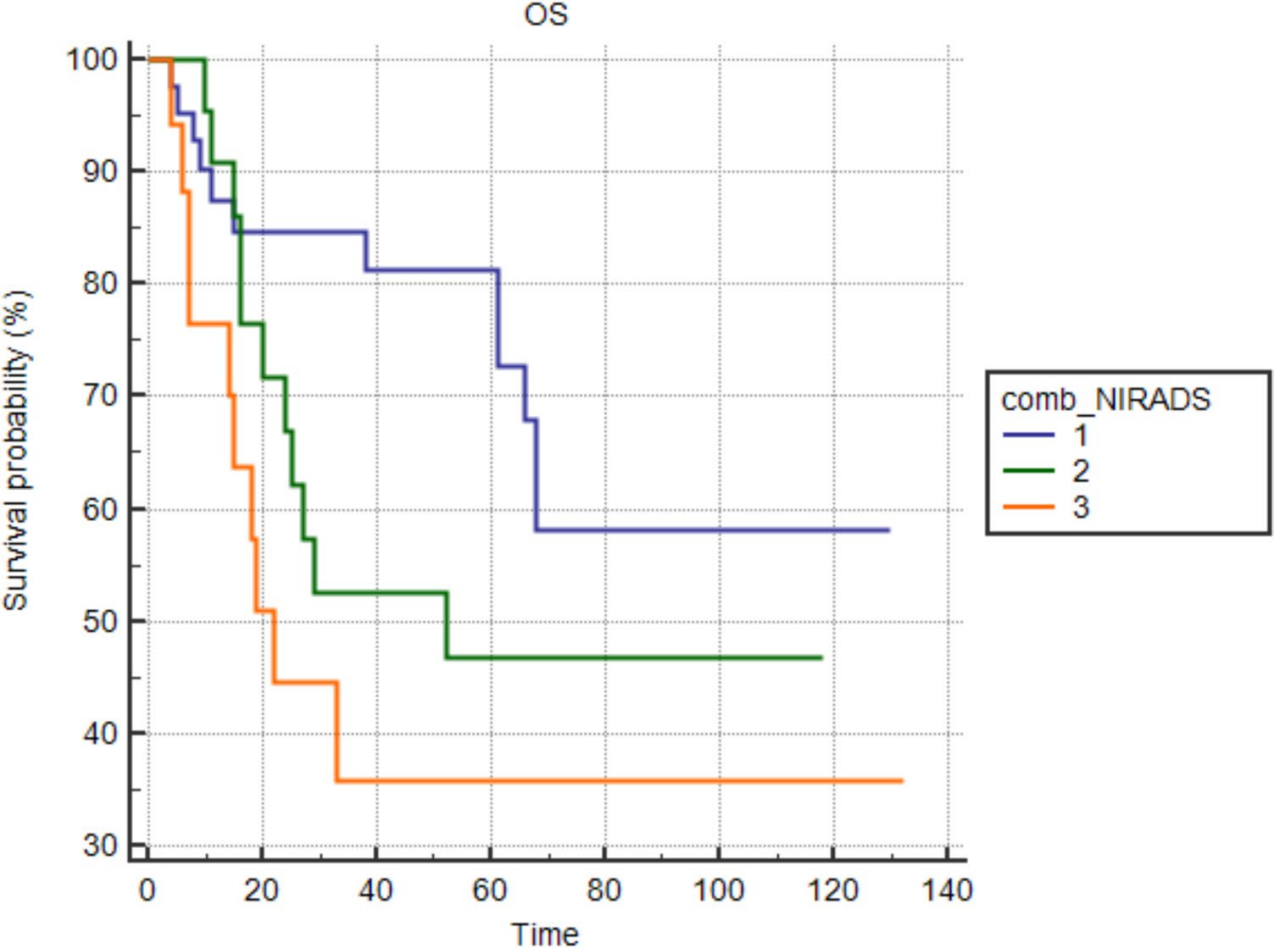
Overall survival (OS) by combined NI-RADS categories

Figures 5 and 6 visually depict DFS and OS stratified by combined NI-RADS categories, underscoring the predictive value of the NI-RADS system for survival outcomes.

## Discussion

This study highlights the prognostic significance of early post-operative NI-RADS scores, in conjunction with conventional factors, for high-risk oral cavity squamous cell carcinoma (SCC) patients undergoing post-operative radiotherapy (PORT). The cohort of 84 patients, with a median age of 62 years and a predominance of males, mirrors the typical demographics of oral cavity cancer. The most common tumor locations were the tongue (48.2%) and buccal mucosa (21.2%). Consistently with other reports in the literature [12] recurrence was observed in 29.4% of patients, primarily as nodal recurrences (16.5%).

Our findings extend earlier surveillance-phase evaluations of NI-RADS. Krieger et al. [11] reported the initial performance of NI-RADS for residual/recurrent HNSCC during post-treatment surveillance, whereas we examined a shorter, early post-operative window before PORT planning in a homogeneous high-risk OCSCC cohort. Dinkelborg et al. [15] specifically studied post-surgical OSCC and found strong discriminatory performance of NI-RADS for recurrence at both primary and nodal sites, consistent with the direction of our results. Additional reports assessing NI-RADS or related post-treatment response frameworks [12–14, 16] collectively support a role for standardized imaging categories to stratify recurrence risk; our data indicate this value might extend to the early post-operative interval.

The study confirmed the importance of traditional prognostic factors, such as nodal involvement and tumor stage, in determining outcomes. However, early NI-RADS scores provided additional predictive value beyond these factors. Significant associations were found between early NI-RADS *T* and *N* categories and both regional progression-free survival (PFS) and overall survival (OS). For instance, NI-RADS *T* scores correlated with regional PFS (*p* = 0.001) and OS (*p* = 0.006, Multivariable *p* = 0.01), while NI-RADS *N* scores showed strong associations with regional PFS (*p* ≤ 0.0001) and OS (*p* = 0.0008). This underscores the utility of NI-RADS in stratifying patients at higher risk of recurrence, enabling more tailored treatment approaches. The high interobserver agreement (Weighted Kappa: *T* = 0.837, *N* = 0.855) further supports the reliability of NI-RADS in clinical practice.

A notable finding was the significant proportion of patients classified as early NI-RADS 2 and 3 post-surgery, indicating a higher risk of recurrence. This aligns with previous studies, which have shown that a substantial proportion of locoregional recurrences in oral squamous cell carcinoma can occur very early, with up to 28% of high-risk patients exhibiting disease progression on diagnostic imaging before the initiation of PORT [5]. Because NI-RADS is interpretive and early post-operative changes can mimic disease, we quantified exploratory misclassification proxies (Supplementary Table S2): early *T* FP 24/34 (70.6%) and FN 8/48 (16.7%); early *N* FP 7/18 (38.9%) and FN 10/66 (15.2%); *combined* FP 20/41 (48.8%) and FN 10/43 (23.3%). These data indicate that higher early NI-RADS categories enrich for events but include many post-surgical mimics, while some NI-RADS 1 cases still recur; thus, early NI-RADS should be interpreted in clinical–surgical context and, when management would change, confirmed with short-interval re-imaging and/or image-guided sampling.

Patients in NI-RADS 2–3 had poorer outcomes despite higher radiotherapy doses, suggesting more aggressive biology that may outweigh the benefits of dose escalation alone. This supports evaluating alternative or intensified strategies (e.g., optimized systemic therapy, clinical trials) for these high-risk groups.

Previous studies show that a prolonged interval between surgery and PORT initiation can worsen locoregional control and survival via accelerated repopulation of residual tumor cells [13, 14]. In our cohort, many patients began PORT beyond the recommended 6–9-week window. Although timing was not significantly associated with outcomes, this null finding is susceptible to confounding by indication, selection effects, and immortal-time bias; given the sample size and event counts, we did not perform additional landmark or time-dependent analyses. Accordingly, the PORT-timing observation should be viewed as hypothesis-generating and validated in larger cohorts using prespecified landmark or time-dependent models. Despite the absence of a detected effect here, prolonged time to RT initiation remains a concern—particularly in high-risk patients—and longer post-surgical recovery (17.2 days for those starting within 9 weeks vs 24.8 days for those starting later) likely contributed to delays, reflecting surgical complexity and patient frailty.

Waiting for patients to undergo a diagnostic CE-CT may have further contributed to delays in treatment initiation. A notable workflow limitation was the use of conventional CT simulation without contrast media. Streamlining the imaging and planning process by incorporating CE-CT directly into the RT simulation workflow could help reduce these delays and improve the accuracy of treatment, particularly for patients with complex post-operative anatomy. Implementing such improvements could lead to faster treatment initiation and potentially better clinical outcomes.

This retrospective, single center study is subject to selection bias and missing data, which may limit generalizability. The overall sample was modest (*n* = 84), with small high-risk strata—particularly NI-RADS 3—reducing precision for subgroup and multivariable estimates; these analyses should be considered exploratory. Imaging was dominated by contrast-enhanced CT, with a small MRI subset, precluding robust conclusions about modality-specific performance of early NI-RADS. No a priori power calculation was performed; accordingly, the study was not powered for definitive subgroup comparisons. We therefore emphasize effect direction and consistency over formal hypothesis testing in small strata.

This study has several limitations. Its retrospective design introduces inherent risks of selection bias and missing data. Being conducted at a single center limits the generalizability of the findings. The relatively small sample size, particularly in certain subgroups, may have affected the statistical power of the analyses. The dominance of CE-CT and the small MRI subset limit conclusions about modality effects on NI-RADS reliability early after surgery. Subgroup counts—particularly NI-RADS 3—were small, reducing precision of multivariable estimates; these analyses should be viewed as exploratory. No a priori power calculation was performed; given the final sample (*n* = 84) and small high-risk strata, the study was not powered for definitive subgroup comparisons. We therefore emphasize effect direction and consistency over formal hypothesis testing in small strata. Because subgroup sizes are small, we focus on the direction and reproducibility of effects rather than on statistical significance tests.

Future research should aim to include larger, multi-center cohorts to validate these findings and explore the integration of NI-RADS into personalized treatment strategies.

## Conclusion

The integration of NI-RADS into early post-operative assessment offers a valuable prognostic tool that enhances predictive capabilities beyond conventional factors. This study supports the routine use of NI-RADS in early imaging to better inform treatment decisions and improve patient outcomes in high-risk oral cavity SCC. Future research should focus on validating these findings in larger, multi-center cohorts and exploring the potential of NI-RADS in guiding personalized treatment strategies.

While the time to PORT initiation did not appear to influence outcomes in this study population, reducing delays and optimizing imaging workflows with contrast-enhanced CT simulation could further improve the treatment of high-risk oral cavity SCC patients.
